# Supplementary material for: Cortical modulation of pupillary function: systematic review
Source: PeerJ. 2019 May 7;7:e6882. doi: 10.7717/peerj.6882 (PMC6510220; doi:10.7717/peerj.6882)
Supplement: Supplemental Information 1 — File including the literature review protocol and an example of the PubMed search as appenix. [file peerj-07-6882-s001.doc]

# Online Supplemental File 1 (S1)

**Cortical modulation of pupillary function: Systematic review**

**(Protocol and Appendix)**

Costanza Peinkhofer ^1,2^, Gitte Moos Knudsen ^1,3,4^, Rita Moretti ^2,5^, Daniel Kondziella^1,6^

^1^ Department of Neurology, Rigshospitalet, Copenhagen University Hospital, Copenhagen, Denmark

^2^ Medical Faculty, University of Trieste, Italy

^3^ Neurobiology Research Unit, Rigshospitalet, Copenhagen University Hospital, Copenhagen, Denmark

^4^ Faculty of Health and Medical Sciences, University of Copenhagen, Copenhagen, Denmark

^5^ Department of Medical, Surgical and Health Sciences, Neurological Unit, Cattinara, Trieste, Italy ^6^ Department of Neuroscience, Norwegian University of Technology and Science, Trondheim, Norway

Corresponding author: Daniel Kondziella, MD, PhD, MSC, FEBN

Department of Neurology

Rigshospitalet, Copenhagen University Hospital

DK-2100 Copenhagen

daniel_kondziella@yahoo.com

+45-3545-63688

1. **Background**

The pupillary light reflex is a polysynaptic reflex that requires cranial nerves II and III, as well as central brainstem connections. Light falling into one eye stimulates retinal photoreceptors, bipolar cells and subsequently retinal ganglion cells, whose axons form the optic nerve. Some of these axons terminate in the pretectum of the mesencephalon; and pretectal neurons project further to the Edinger-Westphal nuclei. Then, preganglionic parasympathetic axons synapse with ciliary ganglion neurons which in turn send postganglionic axons to innervate the pupillary constrictor muscles of both eyes.^1^

Although the pupillary light reflex is part of the routine neurological examination, its physiological background is less well-understood than most clinicians are aware of. In addition to the pathways outlined above, there is also a cortical component of pupillary innervation. For instance, emotional responses such as fear and cognitive processes such as decision making and mental arithmetic may produce pupillary dilatation.^2^ Similarly, electrical stimulation of the frontal eye field leads to pupillary dilatation.^3^ However, the exact mechanisms of supratentorial modulation of the pupillary function remain elusive.

1. **Objectives**

The aim of the present systematic review is to identify a) which cortical or subcortical cerebral areas, and b) which behaviors and cognitive processes, modulate pupillary function in humans and non-human primates. Using the PICO approach^4^, we have phrased the following research questions:

- 1. **Primary Objectives**

1. In patient with focal cerebral lesions due to e.g. stroke, traumatic brain injury or brain surgery (P), does involvement of salient cortical and subcortical gray matter areas, including but not limited to the prefrontal eye field, insular cortex and thalamus (I), as compared to healthy controls or neurological patients without such lesions (C), lead to changes of pupillary function, i.e. the light reflex or resting state pupillary diameter (O)?
2. In healthy human subjects (P), do cognitive efforts (e.g, decision making or mental arithmetic) and processing of non-painful emotional stimuli (I), as compared to task-negative and emotionally neutral conditions (C), lead to changes of pupillary function, i.e. the light reflex or resting state pupillary diameter (O)?
   1. **Secondary Objectives**
3. In non-human primates (P), does invasive experimental manipulation (e.g. electrical stimulation) of cortical and subcortical gray matter areas (I), as compared to absence of stimulation (C), lead to changes of pupillary function, i.e. the light reflex or resting state pupillary diameter (O)?

1. In non-human primates (P), do cognitive efforts such as decision making and processing of non-painful emotional stimuli (I), as compared to task-negative and emotionally neutral conditions (O), lead to changes of pupillary function, i.e. the light reflex or resting state pupillary diameter (C)?
2. **Methods**
   1. **Systematic literature review**

For each PICO question a systematic review of the literature using a predefined search strategy will be performed. Pertinent studies will be identified, their eligibility assessed and data relevant to the PICO question extracted.

**Types of studies**

We will evaluate all cross-sectional or longitudinal, retrospective or prospective, observational clinical and research studies as well as interventional trials, including experimental animal work on non-human primates, and reporting on pupillary function as related to modulation by cortical and subcortical lesions or stimulation, as well as modulation by cognitive and emotional processes.

We will include studies published in English and listed in PubMed, EMBASE and Scopus since January 1, 1960. (Non-English literature will be included only in case that an English abstract and a reliable translation of the manuscript into English are available.) Reviews, meta-analysis, case reports, case series and studies with less than 15 patients will be excluded.

*Participants*

*Inclusion criteria*

1. Patients aged ≥18 years with ischemic or hemorrhagic stroke, brain trauma and/or brain surgery or healthy human controls studied in order to correlate pupillary function to cerebral cortical and/or subcortical gray matter areas and/or specific cognitive or emotional cerebral processing related to experimental invasive or non-invasive stimulation
2. Non-human primates with or without cerebral lesions studied in order to correlate pupillary function to cerebral cortical and/or subcortical gray matter areas and/or specific cognitive or emotional cerebral processing related to experimental invasive or non-invasive stimulation

*Exclusion criteria*

Human patients or non-human primate subjects with brain lesion other than cortical or subcortical gray matter areas or ophthalmologic disorders that might render assessment of the pupillary function unreliable (e.g., cerebral midline shift leading to compression of ophthalmic nerve, eye cataracts), human patients with other neurological or psychiatric diseases such as epilepsy, Parkinson, Alzheimer, depression or schizophrenia ; but, for instance, patients with locked-in syndrome brain-computer interfaces based on pupillary function will be included if their data are compatible with preserved pupillary function

**Index tests**

The index tests comprise neuroimaging (CT, MRI, including functional MRI, PET, SPECT) or post-mortem examination revealing the extent of brain lesions, as well as pupillometry and similar devices used for assessment of pupillary function.

**Interventions**

We will include studies with invasive (e.g. electrical cortical stimulation) or non-invasive (e.g. cognitive and emotional tasks) stimulation of healthy humans, humans with neurological diseases, and non-human primates, addressing the correlation of this stimulation with pupillary function. In addition, observational studies without intervention will be included, if they correlate pupillary function with acquired brain disease involving cortical and/or subcortical gray matter lesions.

**Electronic literature search**

We will search MEDLINE (PubMed), EMBASE and the Scopus for relevant English literature from January 1, 1960 to November 15^th^, 2018. (The search will be updated shortly before submission of the planned manuscript in order to include also the newest references.) The literature search will be supervised by an information specialist from the Copenhagen University Library Service, and the search strategy will be developed in accordance with the clinical question.

We will use the following search terms: “traumatic brain injury”, AND/OR “brain contusion”, “stroke”, “cerebral infarct*”, “intracranial hemorrhage”, “intracranial bleeding”, “subarachnoid hemorrhage”, “brain surgery”, “neurosurgery”, “intracranial EEG”, “electrocorticography”; “cerebral cortex”, “gray matter”, “insular cortex”, prefrontal eye field”, “thalamus”, “basal ganglia”, “caudate nucleus”; “pupil*”, “pupillary light reflex”, “anisocoria”, “pupillary dilation”, “pupillary constriction”, “pupillary diameter”; “pupilometer”, “pupillometry”; “pupil size”; “cognition”, “cognitive”, “emotions”, “attention”, “mental effort”.

Non-English literature will be included only if an English abstract is available and a reliable translation of the manuscript into English possible. Reports exclusively dealing with data on pediatric patients (age <18 years) will not be included. The references of relevant articles will be manually searched to identify additional articles. Further, papers will be cross-referenced using the ‘cited by’ function on PubMed. If necessary, personal communication with authors will be attempted via email or phone in order to obtain additional relevant data. The search strategies (including MeSH headings for searches in PubMed) will be saved and recorded in an appendix.

- 1. **Data collection and analysis**

**Selection of studies**

Titles will be reviewed first, followed by evaluation of the abstracts with titles suggesting that a study might be of relevance. Eligible studies will be identified on the basis of their full text. All potentially relevant articles will be reviewed, confirmed for inclusion. We will use proprietary reference manager software to manage the large number of studies, and we will document the study selection in a detailed flow chart.

**Data extraction and management**

Apart from interventions and outcomes, we will record for human studies 1) journal name, publication date and Vancouver-style reference, 2) study design (e.g. experimental or clinical; cross-sectional or longitudinally), 3) method of recruitment (e.g. prospective or retrospective), 4) study setting, 5) characteristics of the patient population (e.g. age, gender, co-morbidities), 6) intervention (invasive, non-invasive), 7) neuroimaging data, 8) post-mortem (if available), 9) assessment of pupillary function (pupillometry, other device); 10 pupillary function (e.g., changes in pupillary diameter); respectively, for animal data, we will record 1) journal name, publication date and Vancouver-style reference, 2) study design (e.g. cross-sectional or longitudinally), 3) study setting, 6) animal species (e.g. rhesus monkeys), 6) intervention (invasive, non-invasive), 7) neuroimaging data, 8) post-mortem (if available), 9) assessment of pupillary function (pupillometry, other device); 10 pupillary function (e.g., changes in pupillary diameter).

Data will be extracted by the first author and cross-checked by the senior authors; any uncertainties concerning data extraction and interpretation will be resolved by consensus by the senior authors.

**Statistical analysis and data synthesis**

Depending on the results of the literature search and review, we will propose to conduct a meta-analysis on all available numerical data which report on the pupillary function as related to cortical and subcortical brain lesions or cognitive and emotional processes as outlined above. This will be subject to the quality of the studies, study design, and risk of bias.

*Investigations of heterogeneity*

We will attempt to explore possible sources of heterogeneity. These will likely be related to variances in methods of clinical diagnosis, inclusion criteria and heterogeneity in pupillometric evaluation and neuroimaging techniques.

**Assessment of reporting bias**

In order to address possible publication bias or exaggeration of treatment effects in small studies of low quality we may analyze the identified studies using a funnel plot (scatter plot of the treatment effects estimated from individual studies on the horizontal axis against a measure of study size on the vertical axis ^38^), if deemed appropriate.

- 1. **Publication and authorship**

Authorship will be defined according to the Vancouver criteria.

- 1. **Funding**

None

### References

1. Kawasaki A. Physiology, assessment, and disorders of the pupil. Curr Opin Ophthalmol. 1999;10:394-400.
2. Stoll J, Chatelle C, Carter O, et al. Pupil responses allow communication in locked-in syndrome patients. Curr Biol. 2013;23:647-8.
3. Ebitz RB, Moore T. Selective modulation of the pupil light reflex by microstimulation of prefrontal cortex. J Neurosci. 2017;37:5008-18.
4. Schardt C, Adams MB, Owens T, et al. Utilization of the PICO framework to improve searching PubMed for clinical questions. BMC Med Inform Decis Mak 2007;7:16.

**Appendix**

**Full electronic search strategy for Pubmed:**

((((((((((((("Brain Injuries, Traumatic"[Mesh]) OR "Stroke"[Mesh]) OR "Intracranial Hemorrhages"[Mesh]) OR "Neurosurgery"[Mesh]) OR "Neurosurgical Procedures"[Mesh]) OR "Electrocorticography"[Mesh])) AND ((((("Cerebral Cortex"[Mesh]) OR "Gray Matter"[Mesh]) OR "Basal Ganglia"[Mesh]) OR "Thalamus"))) OR (((("Cognition"[Mesh]) OR "Mental Processes"[Mesh]) OR "Attention"[Mesh]) OR "Emotions"[Mesh]))) AND ((((("Pupil"[Mesh]) OR "Reflex, Pupillary"[Mesh]) OR "Anisocoria"[Mesh]) OR "Mydriasis"[Mesh]) OR "Miosis"[Mesh]))) OR ((((((((((((((traumatic brain injury) OR brain contusion)) OR ((stroke) OR cerebral infarct*)) OR (((intracranial hemorrhage) OR intracranial bleeding) OR subarachnoid hemorrhage)) OR ((brain surgery) OR neurosurgery)) OR ((electrocorticography) OR intracranial eeg))) AND (((((((cerebral cortex) OR gray matter) OR insular cortex) OR prefrontal eye field) OR thalamus) OR basal ganglia) OR caudate nucleus))) OR (((((cognition) OR cognitive) OR emotions) OR mental effort) OR attention))) AND (((((((((pupil*) OR pupillary light reflex) OR anisocoria) OR pupillary dilation) OR pupillary constriction) OR pupillary diameter) OR pupilometer) OR pupillometry) OR pupil size)) NOT Medline [SB]) filtered by publication date from 1960/01/01 to 2018/11/15
