# Supplementary material for: Cortical modulation of pupillary function: systematic review
Source: PeerJ. 2019 May 7;7:e6882. doi: 10.7717/peerj.6882 (PMC6510220; doi:10.7717/peerj.6882)
Supplement: Supplemental Information 2 — Checklist of 27 items following PRISMA guidelines. [file peerj-07-6882-s002.doc]

| Section/topic | # | Checklist item | Reported on page # |
| --- | --- | --- | --- |
| TITLE | | |  |
| Title | 1 | Identify the report as a systematic review, meta-analysis, or both. | 1 “Cortical modulation of pupillary function: Systematic review “ |
| ABSTRACT | | |  |
| Structured summary | 2 | Provide a structured summary including, as applicable: background; objectives; data sources; study eligibility criteria, participants, and interventions; study appraisal and synthesis methods; results; limitations; conclusions and implications of key findings; systematic review registration number. | 2, 3: “The review was registered with PROSPERO registration number CRD42018116653” |
| INTRODUCTION | | |  |
| Rationale | 3 | Describe the rationale for the review in the context of what is already known. | 3 “…the exact mechanisms of supratentorial modulation of pupillary function remain poorly understood. Previous (unsystematic) reviews have focus mainly on cognitive aspects such as attention but not on pupillary cortical ” |
| Objectives | 4 | Provide an explicit statement of questions being addressed with reference to participants, interventions, comparisons, outcomes, and study design (PICOS). | 3-4 (see Methods, Objectives) |
| METHODS | | |  |
| Protocol and registration | 5 | Indicate if a review protocol exists, if and where it can be accessed (e.g., Web address), and, if available, provide registration information including registration number. | 3: “The review protocol can be accessed from the online supplementary files (*S1*)”; same as #2 |
| Eligibility criteria | 6 | Specify study characteristics (e.g., PICOS, length of follow-up) and report characteristics (e.g., years considered, language, publication status) used as criteria for eligibility, giving rationale. | 4-5, Protocol :Type of studies, Participants |
| Information sources | 7 | Describe all information sources (e.g., databases with dates of coverage, contact with study authors to identify additional studies) in the search and date last searched. | 4: MEDLINE (PubMed), EMBASE and Scopus;  “January ^1st^ 1960 to November 15^th^, 2018”; |
| Search | 8 | Present full electronic search strategy for at least one database, including any limits used, such that it could be repeated. | Appendix |
| Study selection | 9 | State the process for selecting studies (i.e., screening, eligibility, included in systematic review, and, if applicable, included in the meta-analysis). | See item 6 |
| Data collection process | 10 | Describe method of data extraction from reports (e.g., piloted forms, independently, in duplicate) and any processes for obtaining and confirming data from investigators. | 5: Titles and abstracts were first reviewed. Eligible studies were assessed on the basis of their full text and referenced using Mendeley Software (https://www.mendeley.com). Data were extracted by the first author and checked by the senior author |
| Data items | 11 | List and define all variables for which data were sought (e.g., PICOS, funding sources) and any assumptions and simplifications made. | 4 “The main outcome measure was a change in pupillary function, i.e. either a variation of the pupillary diameter or a difference in the light reflex (e.g. a longer latency period), compared to a baseline value or a control group”;  Protocol |
| Risk of bias in individual studies | 12 | Describe methods used for assessing risk of bias of individual studies (including specification of whether this was done at the study or outcome level), and how this information is to be used in any data synthesis. | Protocol:  “We will attempt to explore possible sources of heterogeneity. These will likely be related to variances in methods of clinical diagnosis, inclusion criteria and heterogeneity in pupillometric evaluation and neuroimaging techniques”;  “In order to address possible publication bias or exaggeration of treatment effects in small studies of low quality we may analyze the identified studies using a funnel plot (scatter plot of the treatment effects estimated from individual studies on the horizontal axis against a measure of study size on the vertical axis), if deemed appropriate.” |
| Summary measures | 13 | State the principal summary measures (e.g., risk ratio, difference in means). | / |
| Synthesis of results | 14 | Describe the methods of handling data and combining results of studies, if done, including measures of consistency (e.g., I^2^) for each meta-analysis. | / |

Page 1 of 2

| Section/topic | # | Checklist item | Reported on page # |
| --- | --- | --- | --- |
| Risk of bias across studies | 15 | Specify any assessment of risk of bias that may affect the cumulative evidence (e.g., publication bias, selective reporting within studies). | See item 12 |
| Additional analyses | 16 | Describe methods of additional analyses (e.g., sensitivity or subgroup analyses, meta-regression), if done, indicating which were pre-specified. | / |
| RESULTS | | |  |
| Study selection | 17 | Give numbers of studies screened, assessed for eligibility, and included in the review, with reasons for exclusions at each stage, ideally with a flow diagram. | 5 We screened 8808 papers in the primary search; two additional publications were manually added. After the exclusion of duplicates, studies with different topic and subjects below 18 years of age, 850 citations were screened for eligibility criteria on an abstract basis. Three-hundred and forty-nine articles were analyzed with a full text review, and 252 studies were included for the final analysis”;  Figure 1 |
| Study characteristics | 18 | For each study, present characteristics for which data were extracted (e.g., study size, PICOS, follow-up period) and provide the citations. | 5-7, Tables 1-2-3 |
| Risk of bias within studies | 19 | Present data on risk of bias of each study and, if available, any outcome level assessment (see item 12). | See item 12 |
| Results of individual studies | 20 | For all outcomes considered (benefits or harms), present, for each study: (a) simple summary data for each intervention group (b) effect estimates and confidence intervals, ideally with a forest plot. | 5-7, tables 1-2-3 |
| Synthesis of results | 21 | Present results of each meta-analysis done, including confidence intervals and measures of consistency. | / |
| Risk of bias across studies | 22 | Present results of any assessment of risk of bias across studies (see Item 15). | / |
| Additional analysis | 23 | Give results of additional analyses, if done (e.g., sensitivity or subgroup analyses, meta-regression [see Item 16]). | / |
| DISCUSSION | | |  |
| Summary of evidence | 24 | Summarize the main findings including the strength of evidence for each main outcome; consider their relevance to key groups (e.g., healthcare providers, users, and policy makers). | 7-9 |
| Limitations | 25 | Discuss limitations at study and outcome level (e.g., risk of bias), and at review-level (e.g., incomplete retrieval of identified research, reporting bias). | 9  “we excluded studies with less than 15 patients;  the tools used to measure pupillary function were not the same across studies; the exclusion criteria regarding previous neurological or ocular pathologies were not always clearly stated” |
| Conclusions | 26 | Provide a general interpretation of the results in the context of other evidence, and implications for future research. | 10 |
| FUNDING | | |  |
| Funding | 27 | Describe sources of funding for the systematic review and other support (e.g., supply of data); role of funders for the systematic review. | 1,protocol:  Financial disclosures: CP, GMK, RM, DK have no financial disclosures |

*From:*  Moher D, Liberati A, Tetzlaff J, Altman DG, The PRISMA Group (2009). Preferred Reporting Items for Systematic Reviews and Meta-Analyses: The PRISMA Statement. PLoS Med 6(7): e1000097. doi:10.1371/journal.pmed1000097

For more information, visit: www.prisma-statement.org.

Page 2 of 2
